# Supplementary material for: WordCommentsAnalyzer: A windows software tool for qualitative research
Source: F1000Res. 2018 Sep 4;7:536. Originally published 2018 May 3. [Version 2] doi: 10.12688/f1000research.14819.2 (PMC6137411; doi:10.12688/f1000research.14819.2)
Supplement: Supplementary file 3 [file f1000research-7-17614-s0002.tgz › b2a79496-4ada-47c4-9682-a99ce867836d.zip!/Eight Iranian Health Journals/Journal of Health System Research _43.docx]

© Journal of Health System Research . This work is licensed under CC BY-NC 4.0 (https://creativecommons.org/licenses/by-nc/4.0/).

Volume & Issue: دوره 7، شماره 2؛ 1390

Title: ارتباط مصرف نوشیدنی‌های شیرین با خطر ابتلا به سرطان: مروری بر مطالعات پیشین

http://hsr.mui.ac.ir/index.php/jhsr/article/view/190

: مقدمه: امروزه سرطان به عنوان يك مشكل عمده سلامت و یکی از علل اصلی مرگ و میر در جهان مطرح است. شيوه زندگي و عادات غذايي، از علل عمده افزايش سرطان مي‌باشد. نوشيدني‌هاي شيرين با توجه به اثراتشان بر هايپر گليسمي، هايپر انسولينمي، كاهش حساسيت انسوليني و چاقی و ارتباط احتمالی آن‌ها با سرطان، مورد توجه محققین قرار گرفته‌اند. با توجه به نبود مطالعه مروري در زمينه ارتباط بین مصرف نوشيدني‌هاي شيرين و خطر ابتلا به سرطان، هدف مطالعه حاضر بررسي اين ارتباط مي‌باشد. روش‌ها: به منظور بررسی مطالعات انجام شده در زمینه ارتباط مصرف نوشیدنی‌های شیرین و ابتلا به سرطان، از جستجو در PubMed و Scirus استفاده شد. یافته‌ها: در اغلب مطالعات آینده‌نگر انجام شده، ارتباط یا ارتباط معنی‌دار بين مصرف نوشيدني‌های شیرین و خطر ابتلا به سرطان مشاهده نشد. در حالی که در اغلب مطالعات مورد شاهدی، مصرف نوشيدني‌های شیرین با افزایش خطر نسبي ابتلا به سرطان همراه بود. در یک مطالعه حیوانی نیز مصرف کولا با افزايش بروز آدنوماي پانكراس همراه بود. نتیجه‌گیری: بر پایه یافته‌های حاصل از مطالعات انجام شده، به نظر می‌رسد که مصرف نوشیدنی‌های شیرین به دلیل محتوای بالای كالري و قند ساده ممکن است موجب افزایش وزن، چاقی و مقاومت انسولینی در افراد گردد و احتمال خطر ابتلا به سرطان را افزایش دهد.

Title: رويكرد توانمندسازي در ارتقای كيفيت زندگي و توان مديريتي بيماران مبتلا به ديابت نوع 2

http://hsr.mui.ac.ir/index.php/jhsr/article/view/222

چكيده روند رو به رشد بیماریهای مزمن غیر واگیر تهدیدی برای سلامت همگان و نوعی اپیدمی جهانی تلقی می شود. این بیماریها متاثر از شرایط فراگیر زندگی و به عبارت بهتر تحت تاثیر سبک زندگی افراد و گروههای اجتماعی شکل می گیرند. دیابت یکی از این بیماریهاست که در کشور ما نیز آمار مبتلایان قابل ملاحظه ای دارد. درمان و کنترل این بیماریها از جمله دیابت با عنایت به ماهیت متفاوت آنها با بیماریهای عفونی و قابل پیشگیری از شیوه های متفاوتی تبعیت می کند. امروزه رویکردهای ناظر به این بیماری عمدتا" رویکردهای متمرکز بر توانمندسازی بیمار است. جند علتی بودن این گروه از بیماریها کنترل آنها را از طریق درمان کلاسیک ناکارآمد می کند و از آنجا که بیمار در رابطه با سبک زندگی خود اطلاعات بسیار بیشتری از درمانگر دارد، مشارکت او با درمانگر در تغییر سبک زندگی به عنوان علت العلل پیدایش و گسترش این بیماری نقشی بسیار کارساز دارد. در رویکرد توانمندسازی بیمار و متخصص دست در دست هم برای کنترل و کاهش پیامدهای بیماری برنامه ریزی و اقدام می نماید. در این رویکرد همه پتانسیل بیمار برای سازگاری با بیماری و تغییر روند آن و گسترش مسئولیت فردی در برخورد با بیماری مورد توجه و تاکید است و این امر موجب می شود که فرآیند درمان نه امری مقطعی بلکه به پدیده ای مستمر تبدیل شود.

Title: کاهش صدای فن و کانال هوا با استفاده از پلنوم (plenum) طبق دستورالعمل ASHRAE

http://hsr.mui.ac.ir/index.php/jhsr/article/view/221

چكيده مقدمه: جهت حذف و کاهش صدا در سیستم‌های تهویه از پیش طراحی و نصب شده، باید میزان صدا را در فضای تحت پوشش سیستم HVAC با استفاده از روش ASHRAE و محفظه‌های پلنوم (Plenum) كنترل نمود. روش‌ها: محفظه به ابعاد 5/1 متر طول، 2/1 متر ارتفاع و 1/1 متر عرض از جنس آهن گالوانیزه به ضخامت 6/0 میلی‌متر با دو دهانه ورودی و خروجی به ابعاد 20 × 40 سانتی‌متر بر اساس نسبت‌های ابعاد مورد بحث در استاندارد اشری ساخته شد. سطح داخلی این محفظه با استفاده از جاذب پشم معدنی با دانسیته حجمی kg/m3 80 و ضخامت 10 سانتی‌متر پوشانده شد. یافته‌ها: میزان کاهش صدا در فرکانس‌های اکتاو شبکه A حداقل 3/2 و حداکثر 2/13 دسی‌بل بوده است. اگر چه این میزان کاهش در فرکانس 250 هرتز دیده می‌شود، ولی به طور کلی بیشترین کاهش صدا در فرکانس‌های میانی و بالا وجود دارد. نتیجه‌گیری: بهترین نتیجه گرفته شده بر اساس استاندارد اشری این است که ابعاد محفظه و ویژگی‌های آکوستیکی مواد جاذب در میزان کاهش صدای محفظه نقش مهمی دارند. در حقیقت می‌توان با تغییر ابعاد محفظه، به نتایج بهتری از کاهش صدای سیستم‌‌های تهویه و کانال هوا دست پیدا نمود. برای حذف بهتر صدای کم فرکانس می‌توان از محفظه بزرگ‌تری استفاده نمود.

Title: بررسی ارتباط کنش شغلی و رفتارهای بهداشتی کارکنان منطقه چهار دانشگاه آزاد اسلامی

http://hsr.mui.ac.ir/index.php/jhsr/article/view/193

چكيده مقدمه: کنش شغلی، علایم جسمی یا روانی ناشی از دریافت یک تنش‌زا در محیط کار است که فرد در مقابله با آن تنش‌زا از منابع سازگاری استفاده می‌نماید. بنابراین با شناسایی نوع تنش، اقدامات آموزشی در جهت کاهش یا حذف هر گونه اثرات آسیب بهداشتی باید انجام گیرد. در این بین، عوامل رفتاری می‌توانند به صورت بالقوه در پیشبرد آموزش و بهداشت مؤثر باشند. این مطالعه با هدف بررسی ارتباط کنش شغلی و رفتارهای بهداشتی کارکنان منطقه چهار دانشگاه آزاد اسلامی انجام شده است. روش‌ها: این پژوهش یک مطالعه توصیفی- همبستگی است که در بین 197 نفر از کارکنان دانشگاه‌های آزاد منطقه چهار که به طور تصادفی خوشه‌ای انتخاب شده بودند، انجام گرفت. ابزار گردآوری داده‌ها پرسش‌نامه‌ای بود که توسط کارکنان تکمیل گردید. یافته‌ها: نتایج نشان داد که بین کنش شغلی و رفتارهای بهداشتی از قبیل رژیم غذایی (001/0 ≤ P)، خواب (003/0 ≤ P) و عواملی نظیر میزان درآمد ماهانه (01/0 ≤ P) ارتباط معنی‌داری وجود داشت. نتیجه‌گیری: نتایج حاصل از این پژوهش بیان داشت کنش شغلی، تأثیر بالقوه زیادی بر روی بهداشت کارکنان دارد و حایز اهمیت می‌باشد. در نظر گرفتن راه کارهایی برای ارتقای بهداشت محل كار می‌تواند به طراحی مجدد مشاغل، كاهش تقاضاهای روان شناختی در شغل و افزایش قدرت تصمیم‌گیری در كارکنان بیانجامد.

Title: تأثیر روش تدریس کارگاهی برآگاهی دانشجویان در ارتباط با بیماری آنفلوآنزای (H1N1)A

http://hsr.mui.ac.ir/index.php/jhsr/article/view/201

چکیده مقدمه: با نظر به آنچه در ارتباط با اهمیت موضوع بیماری آنفلوآنزای (H1N1) A مطرح است و نیز اهمیت موضوع انتخاب روش آموزشی، مقاله حاضر تأثیر روش تدریس کارگاهی را در ارتباط با موضوع بیماری نوپدید همه‌گیری دهنده آنفلوآنزای نوع A مورد بررسی قرار داد. روش‌ها: این مطالعه نیمه تجربی در دانشگاه علوم پزشکی اصفهان در سال تحصیلی 89-1388 انجام شد. 67 نفر از دانشجویان دانشکده بهداشت در مقطع کارشناسی با استفاده از روش نمونه‌گیری تصادفی ساده به عنوان نمونه پژوهشی انتخاب شدند. ابزار جمع‌آوری داده‌ها پرسش‌نامه خودایفای کشوری مقابله با همه‌گیری آنفلوآنزا بود. برای تحلیل نتایج از نرم‌افزار SPSS و روش‌های آماری توصیفی و آزمون‌های آماری استفاده گردید. نتایج: روش تدریس کارگاهی تأثیر معنی‌داری بر افزایش آگاهی دانشجویان نسبت به بیماری آنفلوآنزای نوع A داشت (05/0 > P). دانشجویان دختر و پسر از نظر میزان آگاهی پس از آموزش به روش کارگاهی، اختلاف معنی‌دار داشتند (05/0 > P). نتیجه‌گیری: روش تدریس کارگاهی بر اساس آنچه در این مطالعه به اجرا در آمد، می‌تواند منجر به افرایش کافی در آگاهی دانشجویان در ارتباط با بیماری‌‌‌های عفونی مسری و همه‌گیر شود.

Title: آگاهی، نگرش و عملکرد کارکنان زن شاغل در مراکز بهداشتی درمانی دانشگاه علوم پزشکی اصفهان در خصوص خودآزمایی پستان 1389

http://hsr.mui.ac.ir/index.php/jhsr/article/view/210

چكيده مقدمه: سرطان پستان به عنوان شایع‌ترین سرطان عضوی در زنان، اولین عامل مرگ و میر زنان 45-40 ساله محسوب می‌گردد. تنها راه مبارزه با این بیماری، تشخیص زودرس و به هنگام آن می‌باشد که حدود 90 درصد مبتلایان از این طریق درمان می‌شوند. بهترین روش تشخیص زودرس سرطان پستان غربالگری و از این میان خودآزمایی پستان (BSE) بهترین روش قابل انجام و در دسترس به شمار می‌آید. با توجه به اینکه کارکنان مراکز بهداشتی درمانی، متولیان بهداشت و سلامت هستند، علاوه بر خود- مراقبتی، می‌توانند با جلب مشارکت مددجویان، ارتقای اهداف بهداشتی را فراهم سازند. از این‌رو مطالعه حاضر با هدف تعیین آگاهی، نگرش و عملکرد کارکنان زن شاغل در مراکز بهداشتی درمانی دانشگاه علوم پزشکی اصفهان در خصوص خودآزمایی پستان صورت گرفت.  روش‌ها: این مطالعه از نوع مقطعی بود و بر روی 119 نفر از کارکنان مراکز بهداشتی درمانی شهر اصفهان انجام گردید. نمونه‌گیری به صورت خوشه‌ای انجام شد. با استفاده از پرسش‌نامه KAP آگاهی، نگرش و عملکرد افراد در مورد خود آزمایی پستان سنجیده شد و اطلاعات به دست آمده، با استفاده از روش‌های آماری توصیفی و استنباطی مورد تجزیه و تحلیل قرار گرفت. یافته‌ها: میانگین سنی آزمودنی‌ها 27/7 ± 3/38 سال بود. سطح آگاهی مرتبط با سرطان پستان و انجام خودآزمایی پستان در اکثریت شرکت کنندگان خوب (8/79 درصد) بود و 45/72 درصد افراد نگرش مثبت داشتند. اما در عمل، تنها (5/39 درصد) از شرکت کنندگان خودآزمایی پستان را هر ماه به طور منظم انجام می‌دادند. بین نگرش با مدرک تحصیلی و همچنین بین سن، مدرک تحصیلی و تعداد دفعات شرکت در کلاس‌های بازآموزی با انجام خودآزمایی پستان در زمان مناسب، ارتباط آماری معنی‌داری مشاهده شد (05/0 > P). 6/12 درصد افراد از عملکرد خوبی برخوردار نبودند. نتیجه‌گیری: با توجه به اینکه کارکنان مراکز بهداشتی می‌توانند نقش بسزایی را در ترغیب مددجویان جهت اتخاذ رفتار‌های بهداشتی و پیشگیرانه ایفا کنند، عملکرد ضعیف در این گروه چندان قابل قبول به نظر نمی‌رسد. به همین دلیل توصیه می‌شود مطالعاتی در خصوص شناسایی عواملی که باعث تشویق زنان به استفاده از روش‌های غربالگری می‌گردند، انجام پذیرد و از عوامل شناسایی شده، جهت ترغیب آن‌‌ها برای استفاده از این روش‌ها بهره‌‌برداری شود.

Title: آموزش مبتني بر سازه‌هاي الگوي اعتقاد بهداشتي و كاهش ميزان سزارين در زنان باردار: يك مطالعه‌ی مداخله‌اي

http://hsr.mui.ac.ir/index.php/jhsr/article/view/216

چكيده مقدمه: شهرستان نورآباد ممسني داراي رتبه اول انجام زايمان به روش سزارين در استان فارس است. عمل جراحي سزارين از جمله اعمال جراحي بزرگ محسوب می‌شود و مانند ساير اعمال جراحي بزرگ با عوارضي همراه است. مطالعه حاضر با هدف تعيين تأثير مداخله آموزشي مبتني بر الگوي اعتقاد بهداشتي بر کاهش سزارين زنان باردار این شهرستان در سال 1388 انجام شده است. روش‌ها: در این مطالعه نیمه تجربی، تعداد 100 زن باردار شکم اول تحت پوشش مراکز بهداشتی درمانی شهری شهرستان نورآباد ممسنی که در هفته‌های 30-20 بارداری خود بودند، به طور تصادفی در دو گروه مورد و شاهد طبقه‌بندی شدند. قبل از اجراي برنامه آموزشي، پرسش‌نامه‌اي در ارتباط با سزارين بر اساس سازه‌هاي الگوي اعتقاد بهداشتي، در دو گروه تکميل شد. گروه مورد، مداخله‌ آموزشي را که بر اساس الگوي اعتقاد بهداشتي طراحي شده بود، دريافت کردند. زنان هر دو گروه، بين 8-4 هفته بعد از مداخله، دوباره پرسش‌نامه‌هاي مربوط را تکميل کردند. داده‌هاي جمع‌آوري شده با استفاده از نرم‌افزار 14SPSS و آزمون‌هاي آماري t مستقل، t زوج، Chi-Square،  Wilcoxon and Mann-Whitneyمورد تجزيه و تحليل آماري قرار گرفتند. یافته‌ها: نتايج نشان دهنده‌ وجود تفاوت معني‌دار در ميانگين نمرات آگاهي (001/0 > P)، حساسيت درک شده (001/0 > P)، شدت درک شده (001/0 > P)، منافع درک شده (001/0 > P) و خود-کارآمدي درک شده (02/0 = P) زنان گروه مورد نسبت به زنان گروه شاهد بعد از انجام مداخله بود. در مورد سازه‌هاي موانع درک شده (09/0=  P)، قصد براي انتخاب نوع زايمان (73/0 = P) و عملکرد (24/0 = P)، تفاوت معني‌داري بين دو گروه بعد از مداخله مشاهده نشد. نتیجه‌گیری: برنامه آموزشي مبتني بر الگوي اعتقاد بهداشتي در افزايش آگاهي و نگرش‌هاي زنان باردار در زمينه زايمان، موفق بوده است، ولي با توجه به اينکه در انتخاب نوع زايمان عوامل متعددي دخيل هستند و افزايش آگاهي و نگرش به طور حتم منجر به انجام عملکرد مطلوب توسط- فرد نمي‌شود، استفاده هم‌زمان از الگوهاي ديگري که بر سياست‌گذاري‌هاي بهداشتي و نرم‌هاي انتزاعي و ... تأثير بگذارند، مي‌تواند کارآيي این الگو را افزایش دهد.

Title: ارزیابی کارآیی ماسک تنفسی بر اساس شاخص بیولوژیکی ارتوکرزول در ادرار کارگران در معرض تولوئن

http://hsr.mui.ac.ir/index.php/jhsr/article/view/217

چكيده مقدمه: در بسیاری از صنایع از جمله صنعت تولید رنگ، کارگران در معرض مواجهه با تولوئن قرار دارند. تولوئن جذب شده در كبد متابوليزه می‌شود و به صورت متابوليت‌هاي ادراری از جمله ارتوکروزول دفع مي‌گردد. در برخی صنایع به دلایل مختلف تنها راه ممکن برای کاهش مواجهه، از وسایل حفاظت فردی استفاده می‌شود. هدف این مطالعه، بررسی کارایی یک وسیله حفاظت تنفسی در کاهش میزان مواجهه شاغلین با تولوئن از طریق پایش بیولوژیکی در ادرار می‌باشد. روش‌ها: مطالعه بر روی شاغلین خط تولید در سه صنعت رنگ (17 نفر) صورت گرفت. در شرایطی که شاغلین از وسیله حفاظت تنفسی استفاده نمی‌کردند، میانگین میزان مواجهه تنفسی هر یک از آن‌ها با تولوئن در طی شیفت کاری اندازه‌گیری شد. روز بعد به همان کارگران وسیله حفاظت تنفسی از نوع رسپیراتور نیم صورت تصفیه‌کننده هوا دارای تک فیلتر A1، حاوی زغال فعال مخصوص جذب گازها و بخارات آلی، داده شد. مجدداً میزان میانگین مواجهه تنفسی با تولوئن اندازه‌گیری شد. در پایان شیفت کاری هر دو روز، نمونه ادرار آن‌ها نیز جمع‌آوری شد و جهت تعیین میزان ارتوکرزول به آزمایشگاه منتقل گردید. اندازه‌گیری ارتوکروزول ادراری با متد 8305NIOSH  و نمونه‌برداری و سنجش میزان تولوئن موجود در هوای محیط کار با متد 1501NIOSH ، صورت گرفت. یافته‌ها: بین میانگین مواجهه کارگران با تولوئن در روز اول و دوم از نظر آماری تفاوت معنی‌داری وجود نداشت (956/0 P =)، اما مقایسه ارتوکرزول ادراری پایان شیفت کاری در روزهای عدم استفاده و استفاده از ماسک، تفاوت معنی‌داری را نشان داد (026/0 P =). همچنین مقایسه میانگین تولوئن استنشاقی بر اساس شاخص ارتوکرزول در ادرار شاغلین که با فرمول محاسبه ‌گردید، در روز عدم استفاده و استفاده از وسیله حفاظت تنفسی، تفاوت معنی‌داری را نشان داد (05/0 >P ). با توجه به کاهش 20 درصد تولوئن استنشاقی، میانگین کارایی ماسک حفاظتی بر اساس فرمول‌های مربوطه 6/42 درصد و فاکتور حفاظتی 7/1 به دست آمد. نتیجه‌گیری: فاکتور حفاظتی برای رسپیراتورهای مورد استفاده بر اساس استانداردNIOSH ، 10 می‌باشد. بنابراین بر مبنای نتایج، ماسک مورد آزمون، کارایی لازم جهت کاهش میزان مواجهه با تولوئن را ندارد و برای حفاظت کارگر در مقابل تولوئن نمی‌توان صرفاً از ماسک حفاظتی به عنوان تنها راه ایمن‌سازی استفاده نمود.

Title: بررسی رابطه ابتلا به اختلالات روان‌پزشکی با خصوصیات اپیدمیولوژیک بیماران بستری در بخش روان‌پزشکی بیمارستان نور اصفهان

http://hsr.mui.ac.ir/index.php/jhsr/article/view/218

چكيده مقدمه: اختلالات روان‌پزشکی نوعی بیماری است که افراد از مشکلات عاطفی و احساسی رنج می‌برند که تمامی جنبه‌های زندگی فرد را در بر می‌گیرند. مطالعه این بیماری‌ها در کشورهای مختلف نشان می‌دهد که در میزان شیوع اختلالات روان‌پزشکی اختلاف عمده وجود دارد. همچنین شیوع اختلالات روان‌پزشکی در حال افزایش است و لازم به نظر می‌رسد که رابطه بین ابتلا به این بیماری‌ها و خصوصیات اپیدمیولوژیک بررسی گردد. روش‌ها: این مطالعه به صورت مقطعی است. در این مطالعه پرونده 3935 بیمار بستری در بخش روان‌پزشکی بیمارستان نور اصفهان طی سال‌های 1383 الی 1388 که مبتلا به اختلالات اضطرابی، اختلالات شناختی، اختلالات شخصیت، اختلالات وابستگی و سوء‌مصرف مواد، اختلالات دوقطبی نوع I، اختلالات دوقطبی نوعII ، اختلالات دوقطبی نوع NOS، اختلالات سایکوتیک، اختلالات سایکلوتمیک و اختلالات افسردگی بودند، مورد بررسی قرار گرفتند. توزیع هر کدام از خصوصیات اپیدمیولوژیک بیماران بر حسب نوع اختلالات روان‌پزشکی مشخص شد. عوامل اپیدمیولوژیک مرتبط با هر کدام از اختلالات روان‌پزشکی با استفاده از آنالیز مدل رگرسیون لجستیک، با کنترل اثر بقیه اختلالات روان‌پزشکی آزمون شدند. یافته‌ها: نسبت مردان بستری به زنان 2/1 می‌باشد. میانگین سن بیماران بستری 21/0 ± 06/33 سال است که مردان جوان‌تر از زنان بستری بودند. اختلالات دوقطبی نوع I، سایکوتیک غیرخلقی و افسردگی به ترتیب فراوان‌ترین اختلالات بودند. با افزایش سن، شانس ابتلا به اختلالات افسردگی، شناختی و مصرف مواد افزایش و شانس ابتلا به دوقطبی I و سایکوتیک غیرخلقی کاهش می‌یابد. شانس افراد سیگاری برای ابتلا به دوقطبی I، NOS و اختلال مصرف مواد بیشتر از افراد غیر سیگاری است. شانس این که بیماران با تعداد بستری‌های بالا دارای اختلال اضطرابی، دوقطبی I، II و سایکوتیک غیرخلقی باشند، بیشتر است. نتیجه‌گیری: در مطالعات گذشته شانس مبتلا بودن به سایکوتیک غیر خلقی و دوقطبی برای افرادی که دارای سابقه بستری بودند بیشتر از سایرین به دست آمد که مشابه همین نتیجه، در این مطالعه مشاهده می‌شود. در این مطالعه در اختلالات اضطرابی، دوقطبی و افسردگی شانس ابتلا زنان بیشتر از مردان به دست آمد. همچنین شانس ابتلا به اختلالات مصرف مواد و سایکوتیک غیرخلقی در مردان بیشتر از زنان به دست آمد که مشابه نتایج مطالعات گذشته می‌باشد.

Title: طراحی و بررسی روایی محتوایی و پایایی یک روش پرسش‌نامه‌ای جهت ارزیابی مقدماتی استرس گرمایی در محیط کار

http://hsr.mui.ac.ir/index.php/jhsr/article/view/223

چكيده مقدمه: تنش گرمایی یکی از مهم‌ترین پیامدهای مواجهه با گرما در دنیا است. شاخص‌های موجود ارزیابی تنش گرمایی به دلیل داشتن محدودیت‌های ذاتی و کاربردی برای غربالگری تنش گرمایی در کشور‌های در حال توسعه مناسب نیستند. هدف این مطالعه طراحی و بررسی روایی محتوایی و پایایی یک روش پرسش‌نامه‌ای برای ارزیابی مقدماتی استرس گرمایی بود. روش‌ها: این مطالعه مقطعی در سال 89-1388 در سه مرحله (1) تولید آیتم بر اساس بررسی متون علمی و مصاحبه، (2) ارزیابی روایی محتوایی توسط 9 نفر از متخصصین بهداشت حرفه‌ای و 30 نفر از ارایه دهندگان خدمات بهداشت حرفه‌ای و (3) تحلیل پایایی پیش نویس بر روی 98 نفر از کارگران انجام شد. یافته‌ها: در مرحله تولید آیتم، 40 آیتم شناسایی شد. در ارزیابی محتوایی در سطح متخصصین، 27 آیتم اصلاح، 3 آیتم حذف و 3 آیتم اضافه گردید. در ارزیابی در سطح کارشناسی 19 آیتم اصلاح گردید. در تحلیل پایایی، 15 آیتم، که همبستگی کمتر از 2/0 با کل داشتند، حدف شدند. ضریب Crobcach's alpha برای 25 آیتم باقی مانده برابر 90/0 به دست آمد. نتیجه‌گیری: با توجه به اینکه 25 متغیر قابل اندازه‌گیری با متغیر‌های شاخص تنش گرمایی WBGT و سایر پرسش‌نامه‌ها، نقاط مشترک بالایی داشتند و همچنین کل روش از پایایی بالایی برخوردار بود، چنین استنباط می‌شود که پس از بررسی روایی سازه و معیار می‌توان از پرسش‌نامه برای ارزیابی مقدماتی تنش گرمایی استفاده کرد.

Title: واکنش متقاطع آنتی‌بادی‌های انسانی به آنتی‌ژن مایع کیست هیداتیک (HCF) انسان و موش

http://hsr.mui.ac.ir/index.php/jhsr/article/view/186

چكيده مقدمه: استفاده از آنتی‌ژن خام مایع کیست هیداتیک در تشخیص بیماری هیداتیدوز یکی از روش‌هایی است که با بررسی‌های سرولوژیکی به موقع می‌توان اثرات سودمندی در درمان سریع بیماری ایجاد نمود. تهیه آنتی‌ژن با منشاء انسانی مشکل می‌باشد و از نظر مالی نیز هزینه‌بر است. اما آیا در انسان و موش واکنش متقاطع به آنتي‌ژن‌های مایع کیست وجود دارد یا خیر؟ کدام آنتی‌ژن بالاترین پاسخ آنتی‌بادی انسانی را به خود اختصاص می‌دهد؟ در چه محدوده‌ای، کدام ایمونوگلوبولین برای این پاسخ‌ها ارجح‌تر است؟ این سؤالات موضوع بحث مطالعه حاضر است. روش‌ها: آنتی‌ژن گوسفندی برای آلودگی تجربی سه گروه موش بالب- سی و سوری مورد استفاده قرار گرفت. سرم انسان‌های بیمار جراحی شده برای کیست هیداتیک علیه آنتی‌ژن مایع انسانی و موشی مورد آزمایش قرار گرفتند. آنتی‌بادی تام و زیرکلاس‌های IgG و نیز IgE به روش الیزا اندازه‌گیری شدند. سرم موش‌های ایمن نیز از نظر IgG، HgG2b، IgE و IgGAM مورد آنالیز قرار گرفتند. نتایج با استفاده از آزمون‌های ANOVA و نیز POST HOC مورد بررسی قرار گرفتند. یافته‌ها: IgG و 4IgG انسانی بالاترین میانگین پاسخ را علیه آنتی‌ژن‌های خام انسانی و موشی کیست هیداتیک نشان دادند. واکنش متقاطع در پاسخ انسان به هر دو آنتی‌ژن و نیز در پاسخ سرم موش به هر دو آنتی‌ژن در سطح بالایی وجود داشت ولی علیه آنتی‌ژن موش شدت پاسخ انسان باIgG  تام به مراتب بیشتر از پاسخ آنتی‌بادی انسان به آنتی‌ژن انسانی است. موش‌های سوری آلودگی تجربی به کیست هیداتیک را بیشتر و بهتر از بالب- سی نشان دادند. نتیجه‌گیری: بین دو آنتی‌ژن هم واکنش متقاطع آنتی‌بادی انسانی و هم آنتی‌بادی موشی وجود دارد. آنتی‌ژن موش جایگزین مناسبی در طراحی کیت‌های تشخیصی کیست هیداتیک می‌تواند باشد.IgG  تام و 4IgG بالاترین پاسخ‌ها را در این واکنش‌های متقاطع نشان دادند.

Title: مدل‌سازي عملکرد بيوفيلتر در حذف سولفيد هيدروژن از جريان هوا

http://hsr.mui.ac.ir/index.php/jhsr/article/view/200

چكيده مقدمه: بيوفيلتراسيون يکي از روش‌هاي مؤثر در حذف آلاينده‌های گازی نظير سولفيد هيدروژن از يک جريان هوا مي‌باشد. وجود يک مدل رياضی جهت پيش‌بينی بازده جداسازی می‌تواند در طراحی و بهينه‌سازی اين دستگاه و همچنين مطالعه فرايند بیوفیلتراسيون مفيد واقع گردد. هدف از انجام اين تحقيق، مدل‌سازي رياضي عملکرد بيوفيلتر در فرايند حذف بيولوژيکی سولفيد هيدروژن از جريان هوا می‌باشد. همچنین تأثير پارامترهاي مهمی نظير ضريب نفوذپذيري در بيوفيلم و ضخامت بيوفيلم بر پيش‌بينی نتايج مورد بررسي قرار می‌گیرد. روش‌ها: برای رسيدن به اهداف فوق، معادلات حاکم که در نتيجه به کارگيری قوانين بقای جرم بر روی يک المان در فضای گاز و بيوفيلم می‌باشند، حل گرديد. با حل اين معادلات توزيع غلظت سولفيد هيدروژن در تمامی نقاط گاز و بيوفيلم مشخص شد و با کمک اين توزيع، بازده جداسازی محاسبه گرديد. یافته‌ها: اين مدل قادر به پیش‌بینی تغييرات ضخامت بيوفيلم با زمان، که در اثر رشد باکتري‌ها و همچنين کنده شدن بيومس از سطح بيوفيلم رخ می‌دهد، می‌باشد. نتايج به دست آمده از مدل رياضی توسعه‌يافته با داده‌هاي تجربي توافق خوبي را نشان مي‌دهند. نتیجه‌گیری: نتايج حاصل از مدل رياضی نشان می‌دهند که بازده حذف سولفيد هيدروژن با افزايش ضريب نفوذ، افزايش سطح ويژه پرکن‌ها و کاهش سرعت گاز ورودي زياد می‌گردد. همچنين بازده حذف سولفيد هيدروژن با افزايش ضخامت بيوفيلم، براي مقادير ضخامت پایين بيوفيلم (20-5 ميکرومتر)، بيشتر می‌گردد. اين امر نمايانگر اين نکته می‌باشد که در ضخامت‌های کم بيوفيلم، سرعت واکنش کنترل‌کننده می‌باشد و مقاومت بيوفيلم تأثير چندانی بر نرخ جداسازی ندارد. افزايش بيشتر در ضخامت بيوفيلم (100-20 ميکرومتر) باعث کاهش بازده حذف سولفيد هيدروزن مي‌شود. به نظر مي‌رسد دليل اين امر افزايش اثر تأثير نفوذ بر نرخ انتقال جرم می‌باشد.
